# Comparison of Proton Versus Photon SBRT for Treatment of Spinal Metastases Using Variable RBE Models

**DOI:** 10.1016/j.ijpt.2025.100743

**Published:** 2025-03-05

**Authors:** Sherif G. Shaaban, Michael LeCompte, Hao Chen, Daniel Lubelski, Ali Bydon, Nicholas Theodore, Majid Khan, Sang Lee, Khaled Kebaish, Lawrence Kleinberg, Ted Hooker, Heng Li, Kristin J. Redmond

**Affiliations:** 1 Radiation Oncology and Molecular Radiation Sciences, Johns Hopkins University, Baltimore, MD, USA; 2 Neurosurgery, Johns Hopkins University, Baltimore, MD, USA; 3 Radiology and Radiological Science, Johns Hopkins University, Baltimore, MD, USA; 4 Orthopedic Surgery, Johns Hopkins University, Baltimore, MD, USA

**Keywords:** Proton spine SBRT, Photon spine SBRT, Spine metastasis, RBE-weighted dose

## Abstract

**Purpose:**

Study of proton stereotactic body radiation therapy (SBRT) for spinal metastasis has been limited, largely due to concerns of increased risk of spinal cord injury given the challenges of end of range relative biological effectiveness (RBE). Although the 1.1 RBE constant for proton beam has been adopted for clinical use, data indicate that proton RBE is variable and dependent on technical-, tissue-, and patient factors. To better understand the safety of proton SBRT for spinal metastasis, this dosimetric analysis compares plans using photon robotic techniques and proton therapy accounting for RBE-weighted dose (D_{RBE}).

**Materials and Methods:**

Nine patients with spinal metastasis were selected to be representative of a broad range of complex clinical practice (3 cervical, 3 thoracic, 3 lumbar) that are uniquely challenging to treat with SBRT were identified. Each vertebral level contained a case with paraspinal extension, a reirradiation case, and a case with high-grade epidural disease (Bilsky grade ≥1c) given that such complex cases in current practice often require target volume under‐coverage with photon SBRT (PH-SBRT) in order to meet organ at risk (OAR) dose constraints. All selected patients were treated with PH-SBRT using a robotic system to a prescription dose of 30 Gy in 5 fractions despite our institutional preference for further dose escalation, because further dose escalation was not feasible in the original planning process while keeping normal tissues below acceptable dose constraints. To see if superior target coverage could be achieved with proton treatment, comparative intensity modulated proton therapy (IMPT) plans were generated with the same prescription dose as what was clinically delivered using the 1.1 RBE constant. Dose escalated IMPT plans were then generated to 45 Gy(RBE) in 5 fractions. Variable RBE models (Carabe, McNamara, and Wedenberg) were then utilized to generate RBE‐weighted dose D_{RBE} distribution for 30 Gy(RBE) and 45 Gy(RBE) plans using the α/β value (which was 3.76 in this study), physical dose, linear energy transfer (LET) value, and dose per fraction parameters. Proton plans used the robust optimization parameters of ±3.5% range and 2-mm setup uncertainties. Planning target volume (PTV) coverage and OARs sparing were compared using the Wilcoxon signed-rank test.

**Results:**

Planning target volume coverage was significantly improved when comparing PH-SBRT at 30 Gy in 5 fractions (median: 25 Gy) to IMPT at 30 Gy[RBE] in 5 fractions (median: 30.3 Gy[RBE], *P* = .02) and 45 Gy(RBE) in 5 fractions (median 35.6 Gy[RBE], *P* = .001). Maximum dose of the spinal cord (cord Dmax) was significantly lower with IMPT at 30 Gy(RBE) (median: 17.6 Gy[RBE], *P* = .04) and 45 Gy(RBE) (median: 16.1 Gy[RBE], *P* = .04) compared to conventional plan at 30 Gy (median: 18 Gy). Spinal cord expansion (cord + 2 mm) maximum dose did not change in both photon (median 21.5 Gy) and proton plans (median 22.5, *P* = .27). Other OARs were better spared with the same and dose-escalated proton plans. No difference was seen in cord Dmax when comparing the PH-SBRT at 30 Gy to D_{RBE} at 30 and 45 Gy(RBE) using Carabe-, McNamara-, or Wedenberg models. However, for spinal cord expansion (cord + 2 mm), there was significant difference between PH-SBRT and D_{RBE} at 30 Gy(RBE) and 45 Gy(RBE) in 5 fractions using Carabe- (median: 25.4 Gy[RBE], *P* = .002), McNamara- (median: 25.1 Gy[RBE], *P* = .003), or Wedenberg (median: 24.8 Gy[RBE], *P* = .0001) models. The average increase in the spinal cord expansion maximum dose using these models compared to the fixed RBE plans was 5.3%.

**Conclusion:**

We report the first dosimetric analysis of proton SBRT for spine metastasis using variable RBE dose models. Compared to photon SBRT, IMPT may provide improved target coverage and better spare adjacent OARs, though fixed RBE models can underestimate the maximum dose to adjacent OARs. Future prospective studies are needed to validate these results.

## Introduction

Incidence of spinal metastases is approximately 40% from solid malignancy origin, with two-thirds arising from breast, prostate, and lung carcinoma.^1^ Optimizing local control (LC) of spinal metastases is critical as uncontrolled lesions may be associated with pain, neurological deficits resulting from compression of the spinal cord or cauda equina, or instability.^2^ Conventional palliative external beam radiotherapy (EBRT) has traditionally been used for the treatment of painful metastases with reported overall pain response rates of 60% to 80%.^3^, ^4^ However, the treatment was delivered with low radiation doses with reported rates of retreatment up to 20%.^5^

With the evolution of oligometastatic disease concept, in addition to improved overall survival (OS) by advances in systemic therapy, the goals of care have changed from short-term palliation to long-term symptom management and prevention of disease progression. As such, dose-intensified stereotactic body radiation therapy (SBRT) has been widely adopted to achieve a longer duration of pain response and long-term prevention of neurologic deficits from tumor progression.^5^

An increasing body of evidence reiterates the importance of dose escalation in optimizing tumor control. The recent High Dose per Fraction, Hypofractionated Treatment Effects in the Clinic (HyTEC) group paper reported the tumor control probability of spinal metastases treated with SBRT with different fractionation schemes.^6^ They reported 2-year local control (LC) of 72% with 30 Gy in 5 fractions. Whereas a dose escalation to 45 Gy in 5 fractions improves LC to 95%.^6^

During SBRT treatment planning, it is common to compromise the coverage of the tumor in order to meet normal tissue constraints of the adjacent critical structures including, most importantly, the spinal cord and cauda equina. As such, planning target volume (PTV) coverage of at least 85% to 90% is generally considered acceptable when delivering ablative doses to the tumor. However, in spite of the sharp radiation dose fall-off typically associated with spine SBRT, achieving even 85% coverage with dose-escalated prescription doses is challenging in certain situations, such as in patients with extensive paraspinal extension, reirradiation cases, and cases with high-grade epidural disease (Bilsky grade ≥1c).

Proton beam therapy has been implemented clinically over the past decades due to its unique physical and biological characteristics of dose deposition at the end of the beam and the absence of exit dose referred to as the Bragg peak.^7^ Proton SBRT has been explored to further develop the principle of high dose to limited treatment volumes and sparing normal tissues around benefiting from these known unique properties.^8^ Several dosimetric studies in lung cancer and hepatocellular carcinoma (HCC) have shown a significant reduction in dose to surrounding organs at risk (OARs) with proton SBRT relative to the conventional photon SBRT (PH-SBRT).^9^, ^10^, ^11^, ^12^, ^13^, ^14^

Specific to spine SBRT, a dosimetric study evaluated the feasibility of using passive scattered proton therapy for the treatment of spinal metastases.^15^ They concluded that proton treatment is a feasible approach with 2 opposed lateral beams for treatment of the upper cervical spine and 5-beam arrangement (posterior, posterior obliques, and posterior oblique patches) for the other spinal levels.^15^ Reif et al,^16^ compared single fraction SBRT plans for spinal metastases using intensity-modulated radiation therapy (IMRT), carbon ion RT, and protons. They found comparable (PTV) coverage for all techniques. However, the dose to the cervical and lumbar spinal cord was significantly lower with carbon RT (4.3 Gy, 9.3 Gy) and with protons (8.1 Gy,10.5 Gy), than IMRT (10.7 Gy, 15.5 Gy). Interestingly, they reported that the treatment times for particle RT were considerably shorter than IMRT (6-7 min versus 12-14 min).^16^

However, these studies did not consider the biological aspect of proton therapy known as relative biological effectiveness (RBE). Proton therapy has been planned and delivered clinically using the assumption that protons RBE relative to photons is 1.1. This assumption ignores strong experimental evidence that suggests the RBE varies along the treatment field, i.e. with linear energy transfer (LET), tissue type, and depth.^17^, ^18^, ^19^, ^20^, ^21^, ^22^, ^23^

Several D_{RBE} models have been proposed to evaluate factors influencing protons RBE that account for range uncertainty.^24^, ^25^, ^26^ These models predicted that the RBE increases with increasing LET and decreases with increasing (α/β) and dose per fraction.^24^, ^25^, ^26^ A Better understanding of D_{RBE} is crucial to help improve proton therapy outcomes and mitigate associated acute and chronic toxicities.^27^, ^28^, ^29^, ^30^ This is particularly important in the context of delivering ablative radiation doses in close proximity to the spinal cord given the irreversible nature and life-altering morbidity associated with a spinal cord injury.

To this end, this study represents the first dosimetric analysis of proton SBRT for spinal metastasis using D_{RBE} dose models comparing photon robotic techniques and proton therapy.

## Methods

### Treatment planning and dosimetric comparison

Nine patients with spinal metastasis were selected to be representative of a broad range of complex clinical practice (3 cervical, 3 thoracic, 3 lumbar) that are uniquely challenging to treat with SBRT were identified. Each vertebral level contained a case with high-grade epidural disease (Bilsky grade ≥1c), a reirradiation case, and paraspinal extension (Figure), given that such complex cases in current practice often require target volume under‐coverage with PH-SBRT in order to meet OAR dose constraints. The study was approved by the Institutional Review Board, which approved a waiver of informed consent because the study posed minimal risk to study participants.

**Figure:**
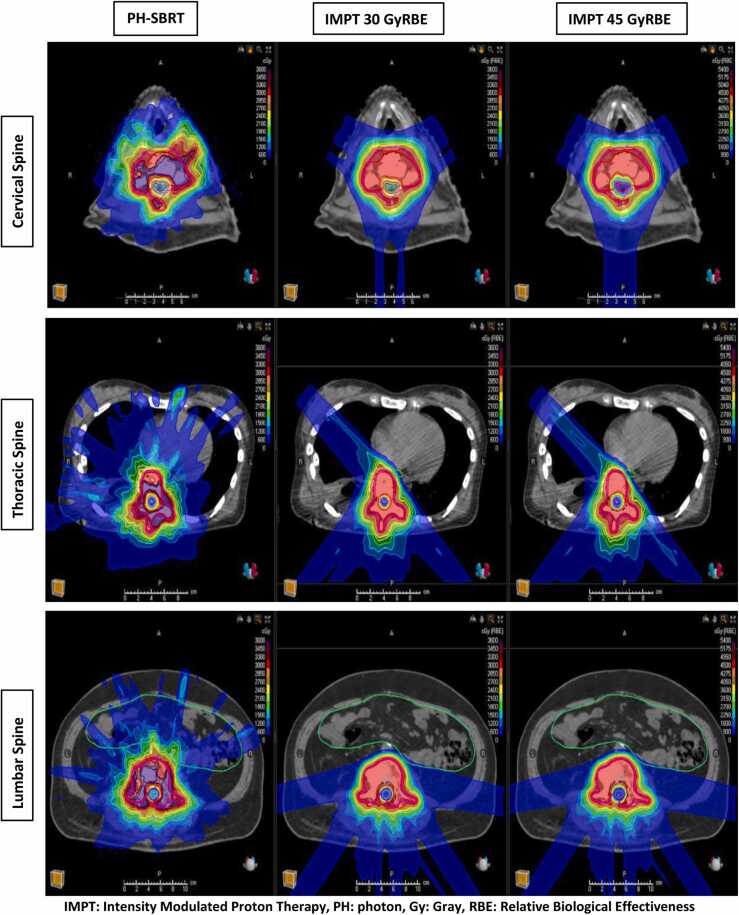
Representative treatment planning slides of the study cohort showing dose distribution of 3 techniques; photon 30 Gy (left), IMPT 30 GyRBE (middle), and 45 IMPT 45 GyRBE (Right). Excess dose spillage to surrounding normal tissues is noted with photon plans.

In clinical practice, all selected patients were treated with PH-SBRT using a robotic system to a prescription dose of 30 Gy in 5 fractions despite our institutional preference of dose escalation, because further dose escalation was not feasible in the original planning process while keeping normal tissues below acceptable dose constraints. The original SBRT plan was performed with CyberKnife (CK) (Sunnyvale, CA). All targets and OAR contours were exported into RayStation treatment planning system Version 12A (RaySearch Laboratories, Stockholm, Sweden) and verified by the attending radiation oncologist. Comparative intensity modulated proton therapy (IMPT) plans were generated with the same prescription dose as what was clinically delivered. Dose escalated IMPT plans were then generated to 45 Gy (RBE) in 5 fractions.

All IMPT plans contain at least 5 coplanar beams to achieve more homogeneous dose distributions while minimizing range uncertainties with expected higher LET and RBE at the distal end of beams to avoid potential injury of surrounding normal tissues. PTV coverage and OARs sparing were compared using the Wilcoxon signed-rank test. PTV was already present for all photon plans. To compare PTV coverage, we created PTV for the proton plans by adding a margin to the clinical target volume (CTV) to account for range and setup uncertainties. Robust optimization was used, with a 3.5% range and 2-mm setup uncertainties, to ensure coverage of at least 90% of the PTV with at least 100% of the prescription dose (V100% ≥ 90%). The spinal cord expansion margin (2 mm) was determined according to the institution’s guidelines. The RBE of 1.1 was used during all IMPT plan optimization. The SBRT plan with 30 Gy was compared with the IMPT plan with 30 Gy(RBE) and 45 Gy(RBE) doses. A fixed 1.1 RBE model was used for plan comparison.

### RBE‐weighted dose (D_{RBE}) Models

With the use of Raystation Version 12A (Stockholm, Sweden) in this study, the dose-average LET was obtained. We included LET from primary and secondary protons with the exclusion of LET from heavier fragments. We then used specific scripts, provided by the vendor, to be able to utilize the variable D_{RBE} models; Carabe,^24^ McNamara,^25^ and Wedenberg.^26^ These models are developed to allow for the estimation of variation in biological effectiveness using the α/βvalue (which was 3.76 in this study), physical dose, LET value, and dose per fraction parameters.

The RBE‐weighted dose D_{RBE} was estimated per each voxel based on the fraction dose of the voxel. For the physical dose per fraction, we initially used 1.2 Gy(RBE) that corresponds to 60% of the standard fractionation of 2 Gy(RBE) since the integral dose of proton therapy is approximately 60% lower than photon treatment.^31^ Then, after the generation of IMPT plan of 45 Gy(RBE) in 5 fractions, we found that the maximum dose would go up to 60 Gy (12 Gy per fraction) with the comparative photon plan. Therefore, we also calculated the variation of RBE using 12 Gy(RBE).

## Results

### Treatment planning and dosimetric comparison

For the 30 Gy in 5 fraction plans, PTV coverage was significantly improved with IMPT compared to PH-SBRT (median: 25 Gy [PH-SBRT], 30.3 Gy[RBE] [IMPT], *P* = .002). The maximum dose of the spinal cord (Dmax) was significantly lower with IMPT (median: 30 Gy [PH-SBRT], 17.6 Gy[RBE] [IMPT], *P* = .04). The maximum dose of spinal cord expansion (cord + 2 mm) did not differ in photon (median 21.5 Gy) and proton plans (median 22.5 Gy[RBE], *P* = .27). The mean dose of the esophagus (Dmean) was significantly lower with protons (median: 15 Gy [PH-SBRT], 12 Gy[RBE] [IMPT], *P* = .018). The heart was better spared with protons (median: 10 Gy [PH-SBRT], 1.0 Gy[RBE] [IMPT], *P* = .04). The mean dose of the Kidneys was significantly lower with IMPT (median: 12 Gy [PH-SBRT], 6 Gy[RBE] [IMPT], *P* = .05). Similarly, bowel Dmax was significantly lower with protons (median: 12 Gy [PH-SBRT], 6 Gy[RBE] [IMPT], *P* = .05) (Table).

**Table:** Comparison of target coverage and OARs constraints of conventional versus same and escalated dose of proton SBRT.

Parameter	PH-SBRT (30 Gy) (Median, range Gy)	IMPT (30 GyRBE) (Median, range Gy [RBE])	*P* value
PTV	25 (15.4-30.2)	30 (17.2-30.3)	**.002**
Spinal cord Dmax	18 (8.2-21.3)	17.6 (7.4-23.3)	**.04**
Cord expansion (cord + 2 mm)	21.5 (10.2-27.8)	22.5 (9.6-27.4)	**.27**
Esophagus Dmean	15 (5.7-30.5)	12 (18.0-24.6)	**.018**
Heart Dmax	10 (9-17.9)	1 (0.1-3)	**.04**
Kidney Dmean	12 (11.2-13)	6 (3.9-7.8)	**5.05**
Bowel Dmax	12 (9.8-14.2)	6 (3.4-8.1)	**.05**

Parameter	PH-SBRT (30 Gy) (Median, range Gy)	IMPT (45 GyRBE) (Median, range Gy[RBE])	*P* value

PTV	25 (15.4-30.2)	35.6 (17-46.6)	**0.001**
Spinal cord Dmax	18 (8.2-21.3)	16.1 (6.8-21.1)	**0.04**
Cord expansion (cord + 2 mm)	21.5 (10.2-27.8)	22.2 (8.7-27.7)	.99
Esophagus Dmean	15 (5.7-30.5)	12 (19-26)	**0.02**
Heart Dmax	10 (9-17.9)	1 (0.1-3)	**0.04**
Kidney Dmean	12 (11.2-13)	6 (3.9-7.8)	**0.04**
Bowel Dmax	12 (9.8-14.2)	6 (3.4-8.1)	**0.03**

**Abbreviations:** IMPT, intensity-modulated proton therapy; PH, photon; Gy, gray; Dmax, maximum dose; Dmean, mean dose; PTV, planning target volume.

With the generation of dose-escalated proton plans to 45 Gy(RBE), PTV coverage was also significantly improved compared to photons (median: 25 Gy (PH-SBRT), 35.6 Gy(RBE) (IMPT), *P* = .001). Also, OARs continued to be better spared with protons; cord Dmax (median: 18 Gy (PH-SBRT), 16.1 Gy(RBE) (IMPT), *P* = .04), esophagus Dmean (median: 15 Gy [PH-SBRT], 12 Gy[RBE] [IMPT], *P* = .02), heart Dmax (median: 10 Gy [PH-SBRT], 1 Gy(RBE) (IMPT), *P* = .04), kidney Dmean (median: 12 Gy [PH-SBRT], 6 Gy[RBE] [IMPT], *P* = .05) and bowel Dmax (median: 12 Gy [PH-SBRT], 6 Gy[RBE] [IMPT], *P* = .03). However, the Dmax of spinal cord expansion (cord+2mm) did not differ in photon (median 21.5 Gy) and proton plans (median 22.2 Gy[RBE], *P* = .99) (Table 1).

### D_{RBE} Models

The 3 used models predicted an increase of the biological dose at the end of the range compared with the fixed RBE plan. With the use of 1.2 Gy(RBE), the predicted biological dose was 129.3%, 128.5%, and 135.0%, respectively. Whereas the use of 12 Gy(RBE) resulted in the reduction of the biologic dose to 107.7%, 102.8%, and 101.0%, respectively.

The D_{RBE} to the spinal cord of the proton plan was compared with the PH-SBRT plan. There was no difference seen in spinal cord Dmax when comparing PH-SBRT at 30 Gy in 5 fractions to D_{RBE} at 30 Gy(RBE) in 5 fractions using Carabe- (median: 17.3 Gy[RBE], *P* = .22), McNamara- (median: 17.4 Gy[RBE], *P* = .22), or Wedenberg (median: 17 Gy[RBE], *P* = .08) models. Similarly, no difference was seen when comparing PH-SBRT at 30 Gy in 5 fractions to D_{RBE} at 45 Gy(RBE) in 5 fractions using Carabe- (median: 17.3 Gy[RBE], *P* = .14), McNamara- (median: 17.4 Gy[RBE], *P* = .17), or Wedenberg (median: 17.0 Gy[RBE], *P* = .09) models. For spinal cord expansion (cord + 2 mm), there was significant difference between PH-SBRT and D_{RBE} at 30 Gy(RBE) and 45 Gy(RBE) in 5 fractions using Carabe- (median: 25.4 Gy[RBE], *P* = .002), McNamara- (median: 25.1 Gy[RBE], *P* = .003), and Wedenberg (median: 24.8 Gy[RBE], *P* = .0001) models. The average increase in the spinal cord expansion maximum dose using these models compared to the fixed RBE plans was 5.3%.

## Discussion

Radiation therapy for spinal tumors is challenging due to its proximity to the spinal cord. It is imperative to limit excess radiation to surrounding healthy tissue in order to minimize the risk of injury associated with permanent life-altering neurological deficits. The use of IMPT represents a potential advantage in utilizing highly sophisticated proton beams to be delivered to the tumor layer by layer with millimeter precision. This offers high chances of controlling the tumor while minimizing radiation exposure to nearby organs, particularly the spinal cord.

We present a dosimetric comparison of proton- versus photon-based SBRT for the treatment of spinal metastases across a representative sample of cases for which obtaining optimal target coverage while achieving normal tissue constraints is challenging. IMPT plans provided superior target coverage and OARs sparing relative to PH-SBRT plans. Furthermore, IMPT planning allowed dose escalation to IMPT plans to 45 Gy(RBE) in 5 fractions while allowing superior sparing of normal structures than the original PH-SBRT plans delivered in the clinic.

These findings are clinically meaningful given that this dose escalation is associated with a local control of 95% compared to 72% following 30 Gy in 5 fractions as demonstrated by the recently published HyTEC tumor control probability analyses.^6^ In addition, the most widely used RBE models were utilized to generate RBE weighted dose (D_{RBE}) distribution for 30 Gy(RBE) and 45 Gy(RBE) plans. There was no significant difference in the maximum dose of spinal cord between the modalities, whereas the maximum dose of spinal cord expansion (cord + 2 mm) was modestly higher with proton plans at 30 Gy(RBE) and 45 Gy(RBE). The average increase in the maximum dose using these models compared to the fixed RBE plans was 5.3%.

The clinical use of protons in the treatment of spinal metastases is still limited. There are no current prospective trials evaluating proton SBRT in this setting yet. Dougherty et al^32^ reported their experience with treating patients with spinal tumors using IMPT between July 2015 and October 2019. Eight percent had prior overlapping radiation therapy fields, and 20% were retreatment. The median dose per fraction and number of fractions were 13 Gy (range 10-22 Gy) and 3 Gy (range, 1-5), respectively. The median follow-up was 22.2 months. Local failure-free survival at 1 and 2 years was 60.6% and 40.9%. Overall survival at 1 and 2 years was 79.1% and 73.8%. All patients were assessed during and following radiation therapy for acute and long-term toxicities. Eighteen percent of patients experienced significant acute adverse events in the form of grade 4 pain flare.^32^

There are few dosimetric studies comparing conventional versus proton SBRT plans. These studies showed superior normal tissue sparing with protons.^7^, ^16^ Rief et al^16^ conducted a dosimetric comparison of a single 24 Gy IMRT versus particle therapy (carbon ion and protons) plans. They reported that the dose to the cervical and lumbar spinal cord was significantly lower with carbon RT (4.3 Gy, 9.3 Gy) and with protons (8.1 Gy, 10.5 Gy), than IMRT (10.7 Gy, 15.5 Gy).^16^

Our results agree with the previous study in the context of better sparing of OARs using the same and escalated dose of IMPT relative to photon plans, namely the spinal cord, heart, kidneys, and bowel. This allows dose escalation without added toxicity to normal structures particularly in challenging cases such as those explored here in which target coverage must be substantially compromised to meet normal tissue constraints.

Proton's biological effects depend on the RBE. A fixed RBE value of 1.1 has been adopted for clinical use, although it is widely accepted that this is an oversimplification.^29^, ^33^, ^34^, ^35^, ^36^, ^37^ Proton RBE uncertainty is greatest at the end of their range where the dose rapidly falls off due to significant changes in energy deposition.^38^

We used 3 common variable RBE models^24^, ^25^, ^26^ to generate (D_{RBE}) distribution for IMPT 30 Gy(RBE) and 45 Gy(RBE) plans. The average increase in the maximum dose using these models compared to the fixed RBE plans was 5.3%. This indicates that the fixed RBE models might underestimate the maximum dose to adjacent OARs. As such, biologically optimized treatment plans that can show the biological hot spots and regions of high RBE are strongly needed in practice to help mitigate potential radiation toxicity which is particularly important when considering delivery of ablative radiation doses in close proximity to the spinal cord.^38^, ^39^, ^40^

Overall, the data presented in this manuscript suggest that proton SBRT may be promising in the treatment of spinal metastases for the following reasons: (1) it allows for dose escalation and increased RBE, especially for radio-resistant metastases relative to photons, (2) it increases differential tumor kill due to higher LET and RBE, and (3) it has more immunogenic properties with decreased lymphopenia, relative to photons. Future prospective studies are needed to evaluate the IMPT feasibility, efficacy, indications, and optimal dose.

This study has some notable limitations including (1) limited in vivo RBE models using human tissue for accurate estimation of biological uncertainties of proton therapy,^27^ (2) Absence of robust evaluation and incorporation of biologically optimized treatment plans that would allow for application of dose escalation safely and efficiently with protection of surrounding normal tissues particularly the spinal cord to achieve higher LC, and (3) lack of a special RBE model for spinal SBRT that allows for more accurate estimation of D_{RBE}.

## Conclusion

We report the first dosimetric comparative analysis of proton‐ versus photon SBRT for the treatment of spinal metastasis using RBE weighted dose D_{RBE} models. Compared to photon SBRT, IMPT may provide improved target coverage and better sparing of adjacent OARs, although fixed RBE models can underestimate the maximum dose to adjacent OARs. Future investigations evaluating the feasibility and safety of proton-based SBRT are needed.

## Author Contributions

Sherif G. Shaaban: Methodology, Writing- Original draft, Writing- Review and Editing. Michael LeCompte: Writing- Review and Editing. Hao Chen: Formal analysis, Writing- Review and Editing. Daniel Lubelski: Writing- Review and Editing. Ali Bydon: Writing- Review and Editing. Nicholas Theodore: Writing- Review and Editing. Majid Khan: Writing- Review and Editing. Sang Lee: Writing- Review and Editing. Khaled Kebaish: Writing- Review and Editing. Lawrence Kleinberg: Writing- Review and Editing. Ted Hooker: Writing- Review and Editing. Heng Li: Writing- Review and Editing. Kristin J. Redmond: Supervision, Writing- Review and Editing.

## Declaration of Conflicts of Interest

The authors declare that they have no known competing financial interests or personal relationships that could have appeared to influence the work reported in this paper.
